# Comparative transcriptome analysis between two different cadmium-accumulating genotypes of soybean (*Glycine max*) in response to cadmium stress

**DOI:** 10.1186/s12863-024-01226-w

**Published:** 2024-05-07

**Authors:** Xiaoqing Liu, Hongmei Zhang, Wei Zhang, Qianru Jia, Xin Chen, Huatao Chen

**Affiliations:** Institute of Industrial Crops, Jiangsu Academy of Agricultural Sciences, Nanjing, 210014 China

**Keywords:** Cadmium, Differentially expressed genes, Soybean, Transcriptome

## Abstract

**Background:**

Cadmium (Cd) is extremely toxic and non-essential for plants. Different soybean varieties differ greatly in their Cd accumulation ability, but little is known about the underlying molecular mechanisms.

**Results:**

Here, we performed transcriptomic analysis using Illumina pair-end sequencing on root tissues from two soybean varieties (su8, high-Cd-accumulating (HAS) and su7, low Cd-accumulating (LAS)) grown with 0 or 50 μM CdSO_4_. A total of 18.76 million clean reads from the soybean root samples were obtained after quality assessment and data filtering. After Cd treatment, 739 differentially expressed genes (DEGs; 265 up and 474 down) were found in HAS; however, only 259 DEGs (88 up and 171 down) were found in LAS, and 64 genes were same between the two varieties. Pathway enrichment analysis suggested that after cadmium treatment, the DEGs between LAS and HAS were mainly enriched in glutathione metabolism and plant-pathogen interaction pathways. KEGG analysis showed that phenylalanine metabolism responding to cadmium stress in LAS, while ABC transporters responding to cadmium stress in HAS. Besides we found more differential expressed heavy metal transporters such as ABC transporters and zinc transporters in HAS than LAS, and there were more transcription factors differently expressed in HAS than LAS after cadmium treatment in two soybean varieties, eg. bHLH transcription factor, WRKY transcription factor and ZIP transcription factor.

**Conclusions:**

Findings from this study will shed new insights on the underlying molecular mechanisms behind the Cd accumulation in soybean.

**Supplementary Information:**

The online version contains supplementary material available at 10.1186/s12863-024-01226-w.

## Background

As a non-essential heavy metal in plants, cadmium (Cd) is toxic and widespread present in the environment [1, 2]. Increasing of Cd levels in the arable soil severely limited the crop yield and harmed human health via the food chain [3]. Cd also exerted adverse impacts on various biochemical and physiological activities of plants, such as growth inhibition, oxidative stresses, protein inactivation and disturbance of nutrient uptake [4, 5]. However, plants have developed a sophisticated arsenal of metabolic “weapons” to combat the Cd-induced stresses, including restriction of intake and transportation, immobilization, chelation and sequestration of Cd in vacuoles, efflux Cd from the cytoplasm [6–8].

Accumulation of Cd in plant shoots is closely related to a myriad of physiological processes, including root uptake, vacuoles sequestration, and xylem and phloem translocation [9]. Generally, Cd can enter plants from soil through either apoplasmic pathway or symplasmic pathway [10]. Plants actively acquire Cd mainly through essential elements uptake systems, involving Fe^2+^, Ca^2+^, Zn^2+^, and Mn^2+^ [11–13]. It has also been suggested that high-affinity Cd transporters were involved in Cd uptake in *Thlaspi caerulescens* [14]. After entering the root, Cd is first delivered to the stele via the endodermis [14] and then transported to shoots via the xylem under the driving force of leaf transpiration [10, 15]. Phloem transportation is responsible for further seed or grain Cd accumulation [16, 17].

During the past decade, the continuing expansion of available transcriptional data has led to identification and characterization of the underlying genetic basis behind the above Cd accumulation physiological processes. Such advancement has tremendously enhanced our ability to explore Cd translocation and detoxification in Cd hyperaccumulating plants, such as *Arabidopsis halleri* [7, 18], *Brassica juncea* [19, 20], *Sedum alfredii* [21], and *Noccaea caerulescens* [22–24], as well as cultivated plants like the pea (*Pisum sativum* L.) [25], barley (*Hordeum vulgare* L.) [26, 27], and rice (*Oryza sativa* L.) [28, 29].

Soybean is a protein- and oil-enriched crop which is a main source of essential amino acids of plant food for humans and animals [30]. Generally, soybean is Cd-sensitive and accumulates Cd even at a low concentration of Cd in soils [31], the risk of Cd accumulation in soybean has raised great concerns [32]. Further, soybean genotypes differ greatly in Cd tolerance and accumulation [33, 34]. The Codex Committee on Food Additives and Contaminants proposed a safe upper limit of 0.2 mg/kg Cd in soybean seeds[35]. However, the Cd uptake, translocation, and accumulation processes in soybeans are still mostly unknown. In this research, we examined the Cd content in shoots and roots of two soybean varieties and found that Cd accumulation in these two varieties was significantly different. We employed comparative transcriptome analysis for the roots of the two soybean varieties before and after Cd treatment to elucidate the potential genetic reasons for the different physiological traits. Findings from this study may provide new insight into the molecular-assisted breeding methods for soybeans.

## Results

### The growth and cadmium accumulation of two soybean cultivars under cadmium treatment

**Fig. 1:**
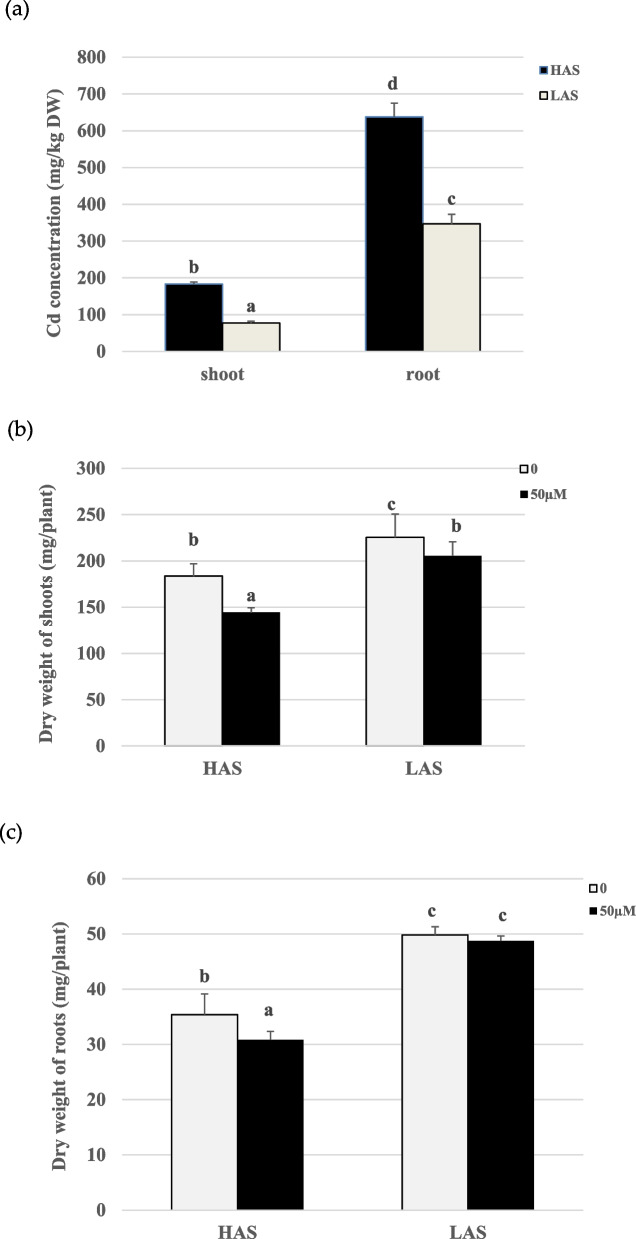
Cd concentrations and dry weight in HAS and LAS soybean varieties under 50 µM CdSO4 treatment. **a** Cd concentrations in shoots and roots in two soybean varieties. **b**, **c** Dry weight of shoots and roots in two soybean varieties. Letters indicate significant differences at the *p* < 0.05 level of the LSD test

After cadmium treatment for 72 h, HAS accumulated more cadmium compared with LAS not only in roots but also in shoots, the accumulated cadmium mainly existed in roots (Fig. 1a). To compare the effects of cadmium on two soybean varieties, we detected the growth parameters. Under both control and Cd treatment, the biomass of LAS was higher than HAS (Fig. 1b, c). The growth of two soybean varieties was all inhibited when treated with 50 µM CdSO_4_ for 72 h. The shoot biomass was significantly inhibited in HAS and LAS after cadmium treatment, decreased by 21.43% and 8.91% separately (Fig. 1b). The root biomass of HAS was more inhibited by cadmium treatment than LAS, decreased by 12.85% and 2.14% (Fig. 1c).

### RNA-Seq analysis

Approximately 18.76 million clean reads were obtained from the soybean root samples after quality control and sequence filtering (Supplementary Material File 1). The GC content of each sample was between 44.25–45.18%, and the average quality score ≥ 30 was 96.04%. The mapping efficiency of the eight samples to the soybean genome (Wm82.a2.v1) was ~ 84.01–86.01%, as shown in Supplementary Material File 2.

### qRT-PCR validation

**Fig. 2:**
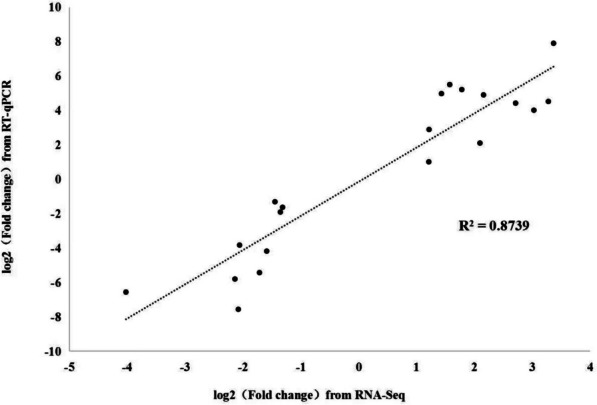
Validation of RNA-Seq data by qRT-PCR of twenty genes in soybean roots after 50 µM CdSO4 treatment in LAS and HAS varieties

Twenty genes (ten genes from LAS, ten genes from HAS) with different expression patterns were randomly selected to preform RT-qPCR (Supplementary Material File 8). The gene expression levels measured by RT-qPCR were compared to those measured by RNA-seq methods, the results showed a highly significant correlation (R^2^ = 0.87) was observed between the RT-qPCR and RNA-seq data sets (Fig. 2), which confirmed that RNA-seq method generated reliable expression data.

### Number of DEGs in different soybean varieties

In order to investigate the molecular mechanisms for different Cd accumulation in the two soybean varieties, transcriptional analysis was conducted. 72 h treatment with 50 µM CdSO_4_ was adopted to explore the response of soybeans to Cd stresses. Transcriptome libraries were created, and sequences from two biological replicates (each for HAS and LAS roots) under control and CdSO_4_ treatments were examined.

**Fig. 3:**
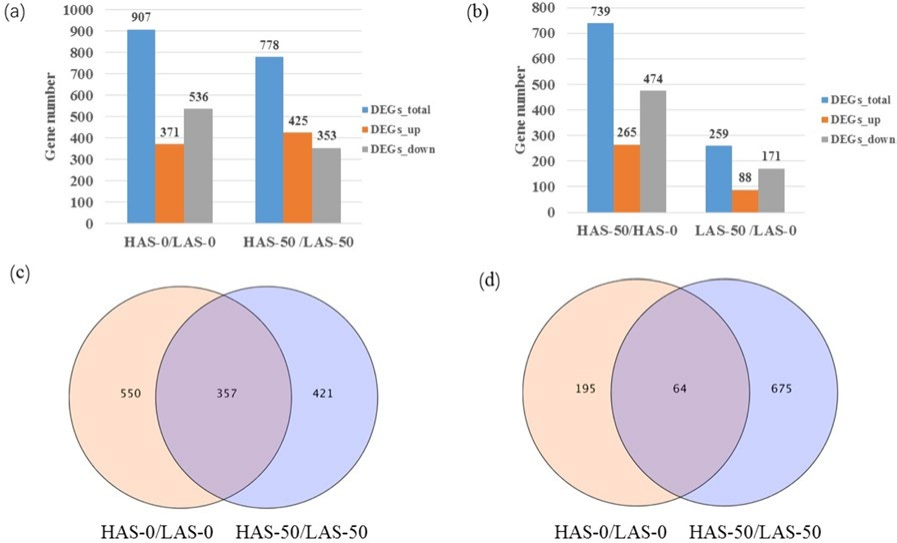
Summary of DEGs. **a** and **b**, number of DEGs between HAS and LAS under 0 or 50 µM CdSO4 conditions. **c** and **d**, Venn diagrams of DEGs in (**a**) and (**c**), respectively

DEGs were identified by comparisons of the FPKM values for each gene between HAS and LAS (HAS-0 versus LAS-0 and HAS-50 versus LAS-50) or between Cd-treated and non-Cd-treated samples of each genotype (HAS-50 versus HAS-0 and LAS-50 versus LAS-0). The results showed that under control conditions, 907 genes (371 up and 536 down) were differentially expressed between HAS and LAS. After Cd treatment, DEGs between HAS and LAS were reduced to 778 (425 up and 353 down), among which 357 genes were common between the varieties, which indicate genetic differences between HAS and LAS (Fig. 3a, c). After Cd treatment, 739 DEGs (265 up and 474 down) were found in HAS; however, only 259 DEGs (88 up and 171 down) were observed in LAS, and 64 genes were common in these DGEs (Fig. 3b, d).

### Gene ontology (GO) analysis of DEGs

**Fig. 4:**
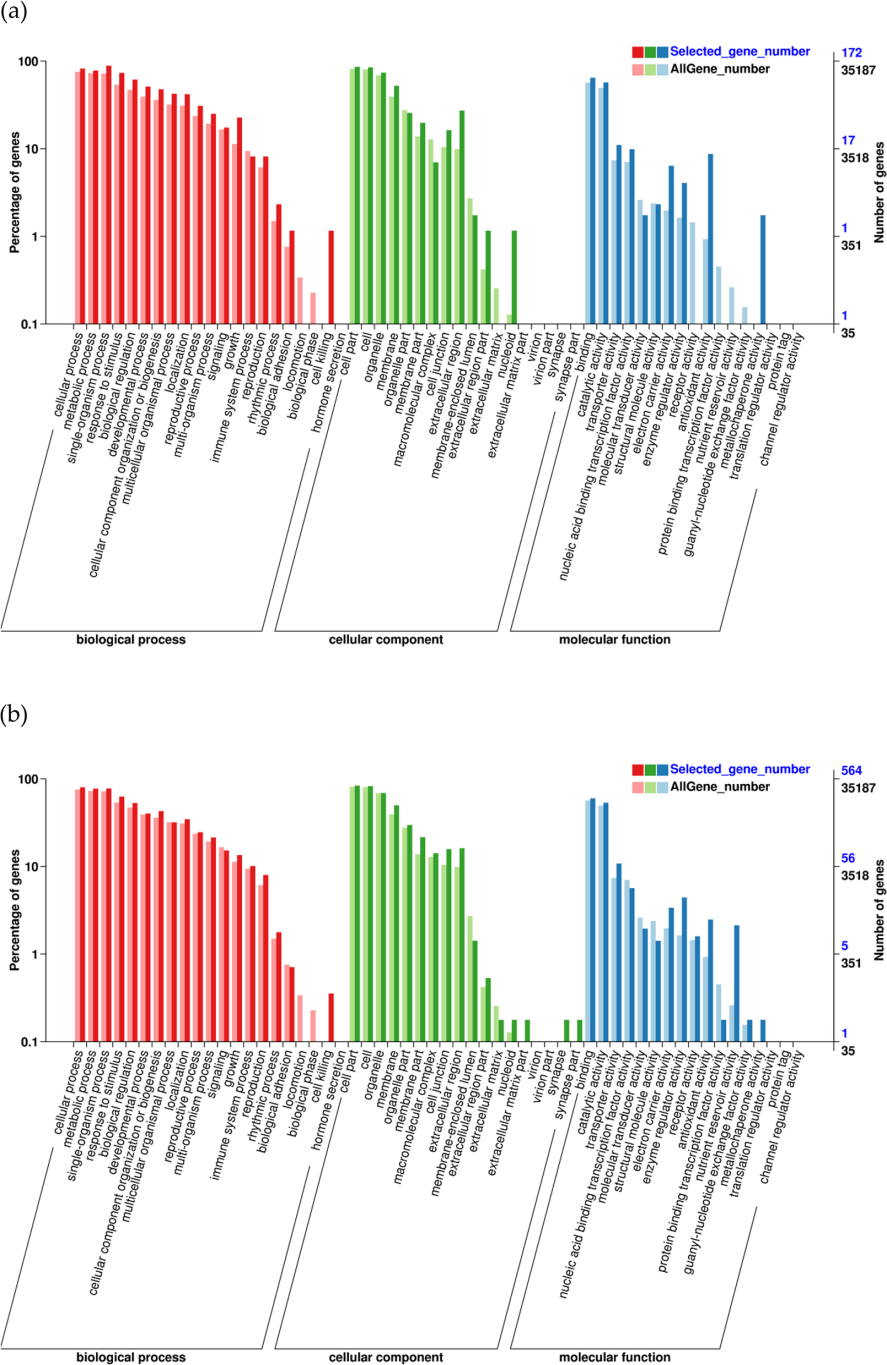
GO enrichment analysis of all DEGs. Genes were assigned into three main categories: biological processes, cellular components or molecular functions. **a** The enriched GO terms of DEGs in LAS after cadmium treatment; **b** The enriched GO terms of DEGs in HAS after cadmium treatment. The y-axis indicates the percentage of DEGs numbers vs. background gene numbers in a given category. Detailed information of analysis of gene GO classification is illustrated in Supplementary Material File 3

To identify the major functional categories represented by the DEGs, GO enrichment analysis was performed (Fig. 4, Supplementary Material File 3). A total of over 21,000 annotated by GO annotation were assigned into three main GO functional categories -biological process, cellular component, and molecular function. For Cd-responsive DEGs, GO items of cellular process, metabolic process, response to stimulus and single-organism process in the biological process category, and cell, cell part and organelle part in the cellular component category, and binding and catalytic activity in the molecular function were enriched in both HAS and LAS(Fig. 4a, b).

### Pathway enrichment analysis of DEGs

**Fig. 5:**
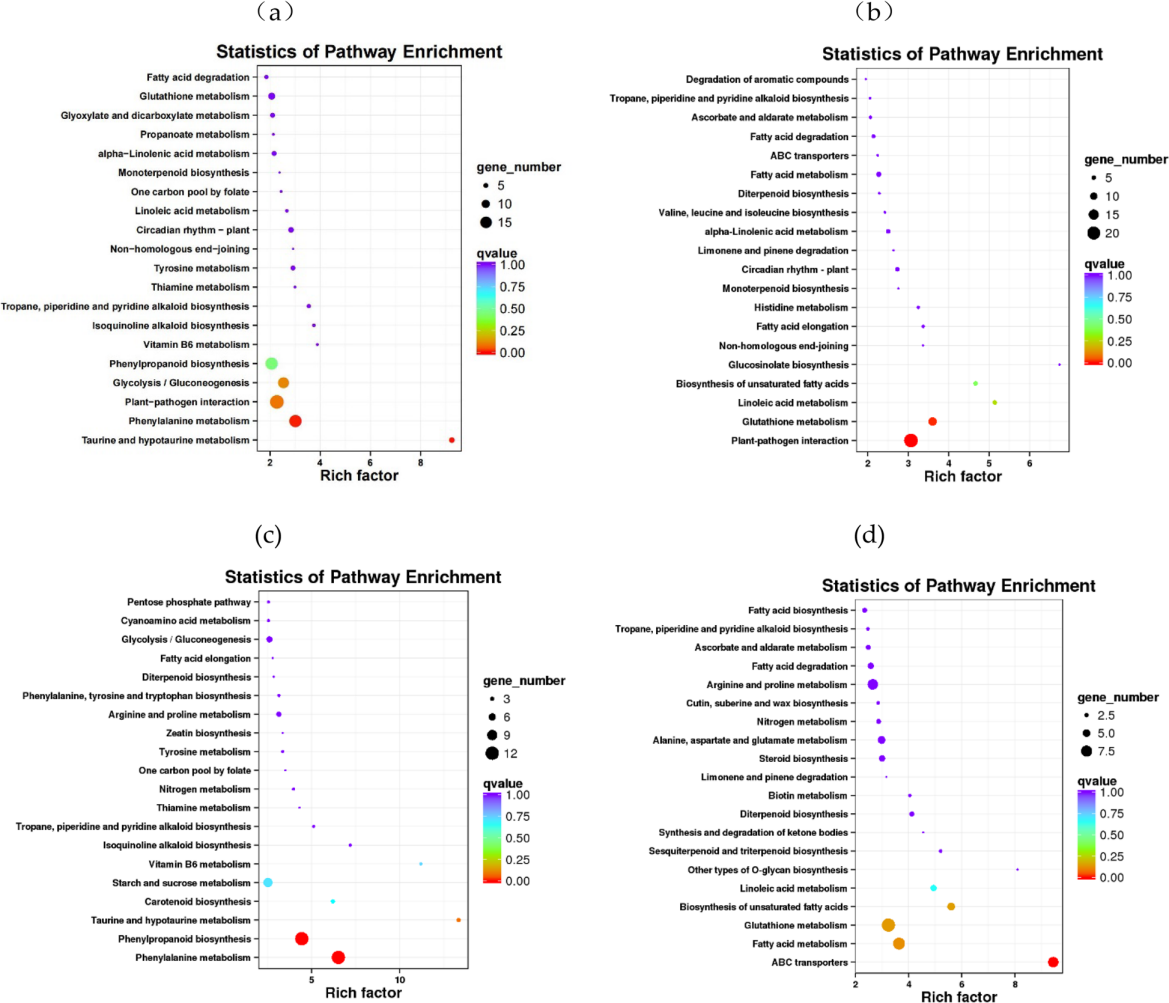
KEGG analysis of DEGs in LAS and HAS without (**a**) or with (**b**) cadmium treatment and DEGs of LAS (**c**) or HAS (**d**) after cadmium treatment. Rich Factor refers to the ratio of the differentially expressed gene number and the number of genes annotated in this pathway and large Rich Factor indicates high degree of enrichment. The area of each colored circle is proportional to the number of genes involved in each pathway, the color indicated the *p* value, and the x-axis is the Rich Factor. Detailed information of KEGG classification is illustrated in Supplementary Material File 4

KEGG pathway enrichment analysis was performed to classify the biological functions of the DEGs by mapping these genes to the reference pathways in the KEGG database. For DEGs between LAS and HAS, pathways of glycolysis/ gluconeogenesis, plant-pathogen interaction, phenylalanine metabolism and taurine and hypotaurine metabolism were enriched under no cadmium treatment (Fig. 5a, Supplementary Material File 4), after cadmium treatment, the DEGs between LAS and HAS were mainly enriched in glutathione metabolism and plant-pathogen interaction pathways (Fig. 5b, Supplementary Material File 4). In LAS, the DEGs were mainly enriched in taurine and hypotaurine metabolism, phenylpropanoid biosynthesis and phenylalanine metabolism pathways under Cd condition(Fig. 5c, Supplementary Material File 4), while in HAS under Cd condition, the DEGs were mainly enriched in biosynthesis of unsaturated fatty acids, glutathione metabolism, fatty acid metabolism and ABC transporters pathways (Fig. 5d, Supplementary Material File 4).

### Phenylalanine metabolism responding to cadmium stress in Low-cadmium-accumulating genotype (LAS)

KEGG analysis showed that phenylalanine metabolism pathway was enriched in LAS after cadmium treatment, suggested that genes involved in this pathway played important role in cadmium tolerance in LAS after cadmium exposure. We analysed the genes differently expressed in this pathway, and found that 10 of 12 DEGs in this pathway encoded one key enzyme- peroxidase, 1 of 126 DEGs encoded aspartate aminotransferase, the rest 1 DEG encoded an uncharacterized protein. (Fig. 5, Supplementary Material File 4).

### ABC transporters responding to cadmium stress in High-cadmium-accumulating genotype (HAS)

Fourteen ABC transporters were found to enriched in HAS after cadmium according to the KEGG analysis. Only 1 ABC transporter was down-regulated, the others were upregulated after cadmium in HAS ((Fig. 5, Supplementary Material File 4).

### Genes involved in heavy metal transport

**Fig. 6:**
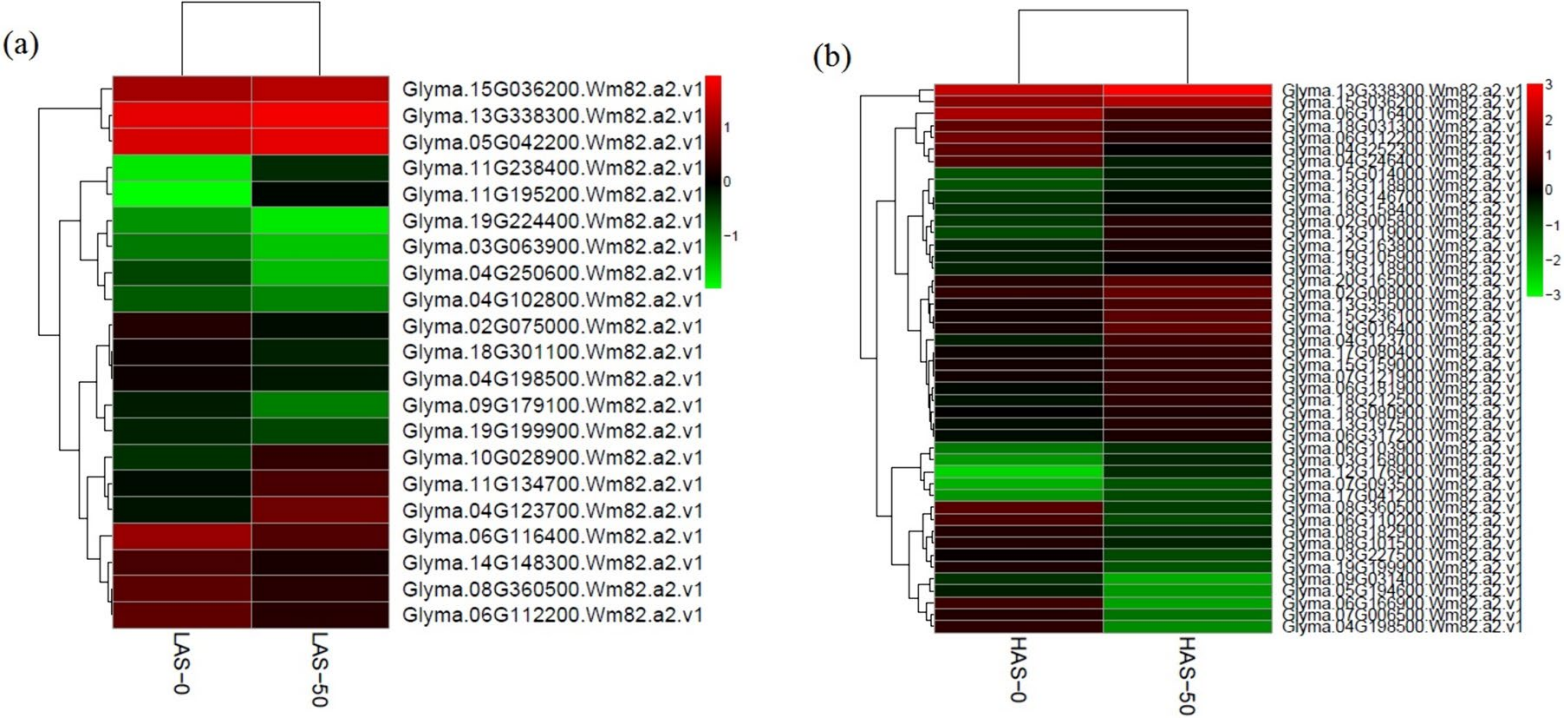
Hierarchical clustering of the transporter genes in LAS (**a**) and HAS (**b**) under Cd exposure. Detailed information of differentially expressed transporter genes is illustrated in Supplementary Material File 5

There were more metal transporter genes differently expressed in HAS-0 versus HAS-50 than in LAS-0 versus LAS-50 (Fig. 6, Supplementary Material File 5), and most of these changes were variety specific. Fourteen ATP-binding cassette (ABC) transporter genes (13 upregulated and 1 downregulated) were found in HAS after Cd treatment. Two zinc-regulated transporter/iron-regulated transporter-like protein (ZIP) zinc transporters (Glyma.13G338300.Wm82.a2.v1 and Glyma.15G036200.Wm82.a2.v1) were found upregulated in both HAS and LAS after Cd exposure

After Cd exposure, two sulfate transporters were upregulated in LAS, while in HAS, only one was upregulated and the other downregulated. Auxin transporters were all downregulated, one in LAS-0 versus LAS-50 and two in HAS-0 versus HAS-50 (Fig. 6, Supplementary Material File 5).

### Differential expression of transcription factors after cadmium treatment

**Fig. 7:**
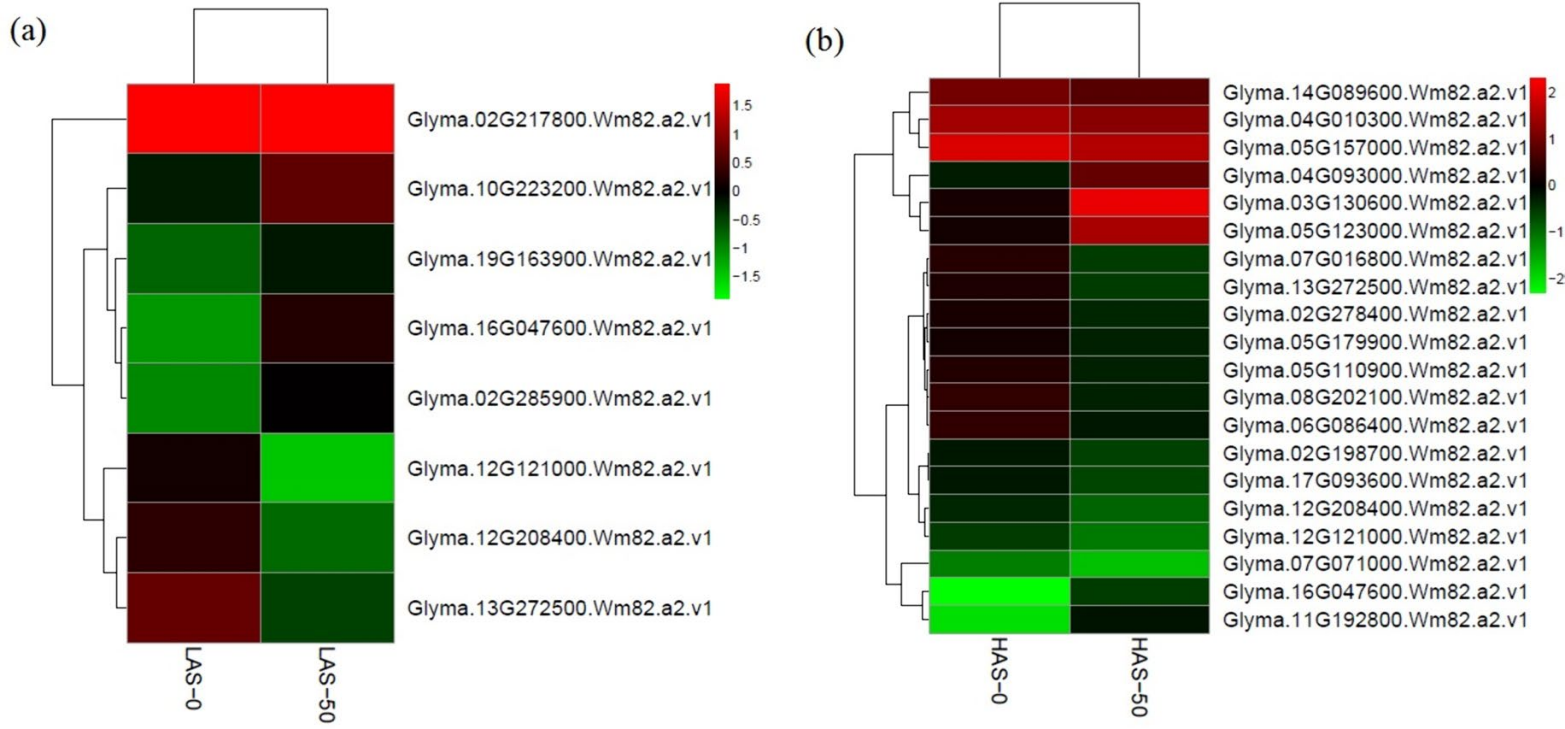
Hierarchical clustering of the transcriptional factors in LAS (**a**) and HAS (**b**) under Cd exposure. Detailed information of differentially expressed transcription factor genes is illustrated in Supplementary Material File 6

Transcription factors (TFs) play important roles in the Cd-responsive gene networks. Several Cd-induced TFs that belonged to different families were identified (Fig. 7a,b, Supplementary Material File 6). One WRKY TF was separately upregulated in LAS-0 versus LAS-50 and HAS-0 versus HAS-50. Three basic leucine zipper (bZIP) TFs were downregulated in HAS-0 versus HAS-50, whereas only one bZIP TF was identified in LAS-0 versus LAS-50(Fig. 7a, b). Four basic helix-loop-helix (bHLH) TFs were differentially expressed in HAS-0 versus HAS-50, two downregulated and two upregulated, while one downregulated bHLH TF was identified in LAS-0 versus LAS-50. Three ethylene-responsive TFs were upregulated in LAS-0 versus LAS-50, while two were identified in HAS-0 versus HAS-50, one upregulated and one downregulated. Further, one downregulated PosF21 probable TF were found not only in LAS-0 versus LAS-50, but also in HAS-0 versus HAS-50, while one upregulated SAC51-like TF, two downregulated E2F TFs, three downregulated GATA TFs, one downregulated EGL TF and one downregulated heat stress TF were found only in HAS (Table 1). Significant differences in metal transporter gene expression were found in LAS-0 versus LAS-50 and HAS-0 versus HAS-50 (Fig. 7a, b, Supplementary Material File 6).

**Table 1:** Differentially expressed genes of the transcription factor family

Transcription factor family	Gene ID	
	LAS	HAS
ER	Glyma.16G047600.Wm82.a2.v1; Glyma.19G163900.Wm82.a2.v1; Glyma.10G223200.Wm82.a2.v1	Glyma.16G047600.Wm82.a2.v1; Glyma.05G179900.Wm82.a2.v1
bHLH	Glyma.02G217800.Wm82.a2.v1	Glyma.11G192800.Wm82.a2.v1; Glyma.03G130600.Wm82.a2.v1; Glyma.14G089600.Wm82.a2.v1; Glyma.05G110900.Wm82.a2.v1
WRKY	Glyma.02G285900.Wm82.a2.v1	Glyma.05G123000.Wm82.a2.v1
bZIP	Glyma.12G208400.Wm82.a2.v1	Glyma.04G010300.Wm82.a2.v1; Glyma.05G157000.Wm82.a2.v1; Glyma.12G208400.Wm82.a2.v1
RF2b	Glyma.13G272500.Wm82.a2.v1	Glyma.13G272500.Wm82.a2.v1
PosF	Glyma.12G121000.Wm82.a2.v1	Glyma.12G121000.Wm82.a2.v1
SAC		Glyma.04G093000.Wm82.a2.v1
E2F		Glyma.02G198700.Wm82.a2.v1; Glyma.17G093600.Wm82.a2.v1
GATA		Glyma.08G202100.Wm82.a2.v1; Glyma.07G016800.Wm82.a2.v1; Glyma.06G086400.Wm82.a2.v1
EGL		Glyma.07G071000.Wm82.a2.v1
Heat stress		Glyma.02G278400.Wm82.a2.v1

## Discussion

After cadmium treatment, two soybean varieties accumulated greatly amount cadmium especially in roots and HAS accumulated more cadmium than LAS not only in shoots but also in roots (Fig. 1a). LAS seems more tolerant to cadmium than HAS. The dry shoot weight of HAS decreased more than the decreased biomass of LAS dry shoot (Fig. 1b). The dry root weight of HAS decreased significantly while there was no influence on the dry root weight of LAS (Fig. 1c). All these results implies the difference mechanism in different soybean cultivars responding to cadmium.

RNA-Seq was conducted to dissect the molecular mechanism behind differential response to Cd stress between these two soybean varieties. Significant genetic differences were found by comparing Cd-responsive DEGs between them. We identified more DEGs in HAS (265 up and 474 down) compared to LAS (88 up and 171 down), indicating a faster response to Cd stress in HAS as compared with LAS. This finding is consistent with the reports of Qian Zhou and colleagues [36] which showed that in pak choi, after Cd treatment, more DEGs were identified in the high-Cd-accumulating genotype compared to the low-Cd-accumulating genotype.

Plants employ an important strategy in Cd detoxification, like antioxidative enzymes. Plant peroxidases (EC 1.11.1.7) are oxidoreductases and have been suggested to be involved in various metabolic steps such as the synthesis of lignin [37, 38], Lignins are particularly important in the formation of cell walls [39], Many studies had reported that heavy metals altered the activity of peroxidases, and the activity of peroxidases which was associated with lignification as a defencing response of roots to Cd [40, 41]. Our results showed that after cadmium treatment, 8 DEGs of peroxidase were high expressed in LAS, which suggested to be crucial for resistance to Cd in LAS.

Among various strategies employed by plants to detoxify Cd, chelation and sequestration are two effective ways of restricting the transportation and circulation of free Cd ions in the cytosol [36]. Reduced glutathione (GSH) plays a vital role in Cd detoxification by conjugation with Cd, a reaction catalyzed by glutathione S-transferases (GST) [2, 42]. After Cd treatment, four GST-encoding genes were upregulated in HAS (Supplementary Material File 7), suggesting that the Cd resistance of HAS depends heavily on the GST-Cd conjugation process. Moreover, GSH could serve as precursors to phytochelatins (PCs), which play important roles in Cd detoxification by forming the PC-Cd complex in plants [36]. Enhancement of PC generation could increase Cd accumulation [43].

It has been suggested that genes involved in Cd efflux and transportation played starring roles in influencing the Cd accumulation ability of different genotypes [43]. These genes are generally categorized into several families based on subcellular location, their sequence specificity, and their carried metals [6]. ABC transporters help transport various substances involved in the response of plants to different environmental stress [44]. For example, it was reported that in rice and *Arabidopsis*, a PDR-type ABC transporter, encoded by *OsPDR9* and *AtPDR8*, was induced by Cd stress and acted as an efflux pump for Cd or Cd conjugates [45, 46]. In our experiment, we found more than ten (mostly upregulated) ABC transporter genes only in the HAS-0 versus HAS-50; this expression may be responsible for the high uptake of Cd in roots and subsequently contribute to the high accumulation of Cd in HAS shoots.

Cd can enter root cells in the form of Cd^2+^ through ZIP transporters, which are capable of transporting a variety of divalent cations, including Cd^2+^, Fe^2+^, Zn^2+^, and Mn^2+^ [47, 48]. In our study, two ZIP transporters (Glyma.15G036200.Wm82.a2.v1 and Glyma.13G338300.Wm82.a2.v1) were upregulated in both genotypes, which indicated that ZIP transporters played vital roles in the uptake of Cd in two soybean genotypes.

Our results revealed that Cd also affected sulfur assimilation. Two sulfate transporters were upregulated in LAS-0 versus LAS-50 only, suggesting that LAS absorbed higher sulfate than HAS under Cd stress. Sulfur assimilation correspondingly activated pathways involved in GSH biosynthesis [49]. GSH functions as an effective antioxidant to mitigate Cd-induced oxidative stresses and plays a vital role in synthesizing PC [42]. Apparently, the sulfur assimilation pathway and GSH synthesis are two important ways of ameliorating the Cd-induced phytotoxicity in LAS.

Auxin, a key plant hormone, which is reported not only to regulate plant growth and development [50] but also in response to cadmiu Cd stress [51]. Auxin transporters, which mediated the transport of auxin, were found to responding to Cd stress not only in LAS but also in HAS. Treatment with the auxin transporter inhibitors increased the Cd sensitivity of WT rice [52]. Our results found that the expression of auxin transporters were decreased after Cd treatment not only in LAS but also in HAS, which suggested that auxin transporter played important roles in soybean responding to Cd stress.

In this research, differential expression of many transporter genes between the two soybean genotypes in response to Cd stress. Together, the overall findings indicate that transporters may play important but different roles in Cd transport and accumulation in LAS and HAS, thus resulting in the corresponding differential Cd tolerance.

TFs participate actively in a wide range of plant stress signaling processes. They belong to different families as diverse as bZIP, WRKY, NAC, ERF (ethylene-responsive factor), and MYB, and are essentially involved in regulating the specific stress-related gene expression under Cd stress [53]. Furthermore, differential expression of several TF genes (WRKY, MYB, ERF, bHLH, and bZIP) was observed in the two soybean genotypes in response to Cd stress, suggesting an important role of TFs in Cd stress response in the soybean, especially in HAS, which exhibited a greater number of differentially expressed TF genes. To decipher the networks of the whole differing Cd-stress-responsive pathway, further analysis of the differentially expressed TF genes is needed. Their potential role in heavy metal tolerance is currently studied by our team.

In summary, we identified two soybean varieties (LAS and HAS) that differentially accumulate Cd and then prepared and sequenced cDNA libraries from untreated and Cd-treated roots. Numerous DEGs were identified in the two soybean varieties under Cd stress. Transcription dynamics of Cd response genes and their related major biological functions were characterized based on GO and KEGG categories. Gene expression analysis suggests that the differential expression of TF and transporter genes is mainly responsible for controlling the contrasting Cd accumulation of the HAS and LAS soybean varieties. Further, research involving in gene function validation will be conducted to clarify the mechanism of its response to cadmium stress.

## Conclusions

Based on the transcriptome sequencing of two soybean varieties (su8, high-Cd-accumulating (HAS) and su7, low Cd-accumulating (LAS)) grown with 0 or 50 μM CdSO4, a total of 18.76 million clean reads from the soybean root samples were obtained. More differentially expressed genes were found in HAS than LAS after cadmium treatment. Differentially expressed genes were mainly distributed in “Plant-pathogen interaction”, “Phenylpropanoid biosynthesis”, “Phenylalanine metabolism”, “Carbon metabolism”, “Starch and sucrose metabolism”, “Glutathione metabolism”, and “Protein processing in endoplasmic reticulum” pathways. DEG clustering and enrichment analysis showed several identified biological processes for coping with Cd stress. Some metal transporters and transcription factors were differently expressed in the two soybean varieties after cadmium treatment. In general, this study revealed new insights on the underlying molecular mechanisms after cadmium treatment, which provides a foundation for further function identification of genes in soybean.

## Methods

### Plant material and Cd treatment

Seeds of two soybean cultivars-su8 (high-Cd-accumulating (HAS)) and su7 (low Cd-accumulating (LAS)) which were selected from Jiangsu Acadmy Agricultural Sciences and suitable for planting in Jiangsu Province were surface sterilized by 2% H_2_O_2_ for 10 min and fully rinsed with deionized water. Thereafter, the sterilized seeds were sown in sterile vermiculite for germination under constant temperature (25 ± 1 °C) and a fixed photoperiod (14:10 h light:dark cycle). After 1 week, similarly sized healthy seedlings of each genotype were transplanted to a half-strength modified Hoagland nutrient solution [54] in a greenhouse under controlled temperature (25 − 28 °C) and a fixed photoperiod (14:10 h light:dark cycle).

When the primary leaves were fully opened, soybean seedlings of the two genotypes were treated with fresh medium with or without CdSO_4_ (final Cd concentration of 50 μM) for 72 h. These conditions represent mild stresses and would not cause visual toxic symptoms for the two genotypes. The experiment was completely randomized with three replicate vessels each with ten seedlings.

For RAN sequencing, after 72 h Cd treatment, shoots and roots of each genotype were separately harvested, followed by wash three times with deionized water. For Cd accumulation determination, washed shoots and roots were oven-dried at 70 °C to a constant weight. Fresh root tissues were frozen in liquid nitrogen (N_2_) and stored in − 80 °C refrigerator for future RNA extraction and subsequent de novo library construction. Roots from four seedlings of each genotype with or without Cd treatment were randomly selected for RNA sequencing. The experiments were performed with three biological replicates.

### Determination of Cd concentration and dry weight

Shoots and roots from three plants of each genotype with or without Cd treatment were oven-dried (70 °C) to a constant weight and weighted, then digested with a solution of extra pure HNO_3_ and HClO_4_ (87:13, v:v) in a microwave. After cooling down, the digester was measured for Cd concentration using FAAS (HITACHI Z-5300, Japan) in accordance with the manufacturer’s instructions. A Certified Reference Material (CRM; GBW-07603, provided by the National Research Center for CRM, China) was used to assess the precision of the analytical procedures for plant material. Data were analyzed using SPSS statistics 17.0 for the analysis of variance (ANOVA) test. Significant differences in Cd concentration between the two genotypes were determined by the least significant difference (LSD) test at *P* < 0.05.

### RNA isolation, RNA-Sequencing (RNA-Seq) library preparation, and sequencing

Total RNA was isolated from frozen and ground root tissue using a plant RNA kit (OmegaBio-Tek, Norcross, GA, USA) according to the manufacturer’s instructions. RNA concentration was assessed using a ND-8000 spectrophotometer (Nanodrop Technologies, Inc., Wilmington, DE, USA), a 2100-Bioanalyzer (Agilent Technologies, Santa Clara, CA, USA), and agarose gel electrophoresis. RNA samples with no observable smearing (a 260/280 ratio above 2.0 and a RNA integrity number greater than 8.0), were collected for subsequent analysis.

The de novo transcriptome analysis was performed by combining three replicate root samples into a single total RNA sample (In total, two samples were prepared for each treatment), which were then sent to the biological company for sequencing.

### De novo transcriptome assembly and annotation

We performed sequencing using an Illumina GAll according to the manufacturer’s protocol with an average sequencing depth of 5.34X. Adapter sequences were eliminated from the raw sequence reads using a FASTX-toolkit. Then, sequence quality was analyzed, and low-quality sequences were removed accordingly using FastQC to obtain clean reads to increase sequence confidence. Clean reads were then aligned to the soybean genome (*Glycine max* Wm82.a2.v1) using Tophat v. 2.0.10. Subsequently, transcriptome assemblies were conducted using Cufflinks, while gene expression levels were calculated as reads per kilobase of exon model per million mapped reads (FPKM) [55]. The differentially expressed genes (DEGs) were identified and screened by DESeq software [56]. In this method, the adjusted *P*-values used the false discovery rate (FDR) of < 0.01 and |log2 (fold change)|> 1 or < -1 as the thresholds for differential gene expression. Meanwhile, the screening process adopted fold changes of the expression levels between different samples as the criteria.

### Validation of gene expression

A set of 20 randomly selected DEGs from the transcriptome analysis (ten genes from LAS, ten genes from HAS) were validated by quantitative Real-time polymerase chain reaction (qRT-PCR) using the same RNA samples used for transcriptome analysis according to [36]. The sequences of the corresponding target genes were obtained from the National Center for Biotechnology Information (NCBI). Primers were designed using Primer Premier 5.0 (Supplementary Material File 8) for qRT-PCR. And genomic DNA was removed using the RNase-free DNase I Set (Omega, USA) according to the manufacturer’s instructions after RNA extraction. First-strand complementary DNA (cDNA) was synthesized from approximately 1 µg of RNA using a reverse transcription kit (BioTeke, China). qRT-PCR was performed using SYBR Green (Bio-Rad) in a LightCycle480 system (Roche).The relative quantification was normalized to the *GmActin* reference gene. The 2^–ΔΔCt^ method was used for data analysis. Each PCR reaction, including the control reaction, was performed in triplicate.

### Statistical analysis

Statistical analyses were performed in Excel and SPSS v17.0 (link/cite SPSS.) The significance threshold between samples was *p* < 0.05, and all results of expression data were indicated as averages ± standard deviations (SDs).

### Supplementary Information

**Supplementary Material 1.**

**Supplementary Material 2.**

**Supplementary Material 3.**

**Supplementary Material 4.**

**Supplementary Material 5.**

**Supplementary Material 6.**

**Supplementary Material 7.**

**Supplementary Material 8.**

## Data Availability

The raw sequence data reported in this paper have been deposited in the Genome Sequence Archive (Genomics, Proteomics & Bioinformatics 2021) in National Genomics Data Center (Nucleic Acids Res 2022), China National Center for Bioinformation / Beijing Institute of Genomics, Chinese Academy of Sciences (GSA: CRA012777) that are publicly accessible at https://ngdc.cncb.ac.cn/gsa [57, 58].

## Abbreviations

Cd: Cadmium
HAS: High-Cd-accumulating soybean variety
LAS: Low-Cd-accumulating soybean variety

## References

[1] He JL, Li H, Luo J, Ma CF, Li SJ, Qu L (2013). A Transcriptomic network underlies microstructural and physiological responses to cadmium in Populus x canescens. Plant Physiol.

[2] Rui HY, Zhang XX, Shinwari KI, Zheng LQ, Shen ZG (2018). Comparative transcriptomic analysis of two Vicia sativa L. varieties with contrasting responses to cadmium stress reveals the important role of metal transporters in cadmium tolerance. Plant Soil.

[3] Uraguchi S, Fujiwara T (2013). Rice breaks ground for cadmium-free cereals. Curr Opin Plant Biol.

[4] Chakrabarti M, Mukherjee A (2021). Investigating the underlying mechanism of cadmium-induced plant adaptive response to genotoxic stress. Ecotoxicol Environ Saf.

[5] Shanying HE, Xiaoe YANG, Zhenli HE, Baligar VC (2017). Morphological and physiological responses of plants to cadmium toxicity: a review. Pedosphere.

[6] Hall JL (2002). Cellular mechanisms for heavy metal detoxification and tolerance. J Exp Bot.

[7] Weber M, Trampczynska A, Clemens S (2006). Comparative transcriptome analysis of toxic metal responses in Arabidopsis thaliana and the Cd2+-hypertolerant facultative metallophyte Arabidopsis halleri. Plant Cell Environ.

[8] Zenk MH (1996). Heavy metal detoxification in higher plants - A review. Gene.

[9] Feng JJ, Jia WT, Lv SL, Bao H, Miao FF, Zhang X (2018). Comparative transcriptome combined with morpho-physiological analyses revealed key factors for differential cadmium accumulation in two contrasting sweet sorghum genotypes. Plant Biotechnol J.

[10] Lu LL, Tian SK, Yang XE, Li TQ, He ZL (2009). Cadmium uptake and xylem loading are active processes in the hyperaccumulator Sedum alfredii. J Plant Physiol.

[11] Lombi E, Tearall KL, Howarth JR, Zhao FJ, Hawkesford MJ, McGrath SP (2002). Influence of iron status on cadmium and zinc uptake by different ecotypes of the hyperaccumulator Thlaspi caerulescens. Plant Physiol.

[12] Pence NS, Larsen PB, Ebbs SD, Letham D, Lasat MM, Garvin DF (2000). The molecular physiology of heavy metal transport in the Zn/Cd hyperaccumulator Thlaspi caerulescens. P Natl Acad Sci Usa.

[13] Perfus-Barbeoch L, Leonhardt N, Vavasseur A, Forestier C (2002). Heavy metal toxicity: cadmium permeates through calcium channels and disturbs the plant water status. Plant J.

[14] Zhao FJ, Hamon RE, Lombi E, McLaughlin MJ, McGrath SP (2002). Characteristics of cadmium uptake in two contrasting ecotypes of the hyperaccumulator Thlaspi caerulescens. J Exp Bot.

[15] Lux A, Martinka M, Vaculik M, White PJ (2011). Root responses to cadmium in the rhizosphere: a review. J Exp Bot.

[16] Kato M, Ishikawa S, Inagaki K, Chiba K, Hayashi H, Yanagisawa S (2010). Possible chemical forms of cadmium and varietal differences in cadmium concentrations in the phloem sap of rice plants (Oryza sativa L.). Soil Sci Plant Nutr.

[17] Uraguchi S, Kamiya T, Sakamoto T, Kasai K, Sato Y, Nagamura Y (2011). Low-affinity cation transporter (OsLCT1) regulates cadmium transport into rice grains. P Natl Acad Sci Usa.

[18] Herbette S, Taconnat L, Hugouvieux V, Piette L, Magniette MLM, Cuine S (2006). Genome-wide transcriptome profiling of the early cadmium response of Arabidopsis roots and shoots. Biochimie.

[19] Farinati S, DalCorso G, Varotto S, Furini A (2010). The Brassica juncea BjCdR15, an ortholog of Arabidopsis TGA3, is a regulator of cadmium uptake, transport and accumulation in shoots and confers cadmium tolerance in transgenic plants. New Phytol.

[20] Fusco N, Micheletto L, Dal Corso G, Borgato L, Furini A (2005). Identification of cadmium-regulated genes by cDNA-AFLP in the heavy metal accumulator Brassica juncea L. J Exp Bot.

[21] Gao J, Sun L, Yang X, Liu JX (2013). Transcriptomic analysis of cadmium stress response in the heavy metal hyperaccumulator Sedum alfredii Hance. Plos One.

[22] Halimaa P, Lin Y, Ahonen VH, Blande D, Clemens S, Gyenesei A (2014). Gene expression differences between Noccaea caerulescens ecotypes help to identify candidate genes for metal Phytoremediation. Environ Sci Technol.

[23] Lin Y, Severing EI, Hekkert BTL, Schijlen E, Aarts MGM (2014). A comprehensive set of transcript sequences of the heavy metal hyperaccumulator Noccaea caerulescens. Front Plant Sci.

[24] Milner MJ, Mitani-Ueno N, Yamaji N, Yokosho K, Craft E, Fei Z (2014). Root and shoot transcriptome analysis of two ecotypes of Noccaea caerulescens uncovers the role of NcNramp1 in Cd hyperaccumulation. Plant J.

[25] Romero-Puertas MC, Corpas FJ, Rodriguez-Serrano M, Gomez M, Del Rio LA, Sandalio LM (2007). Differential expression and regulation of antioxidative enzymes by cadmium in pea plants. J Plant Physiol.

[26] Cao FB, Chen F, Sun HY, Zhang GP, Chen ZH, Wu FB (2014). Genome-wide transcriptome and functional analysis of two contrasting genotypes reveals key genes for cadmium tolerance in barley. BMC Genomics.

[27] Tamas L, Dudikova J, Durcekova K, Halugkova L, Huttova J, Mistrik I (2008). Alterations of the gene expression, lipid peroxidation, proline and thiol content along the barley root exposed to cadmium. J Plant Physiol.

[28] Oono Y, Yazawa T, Kawahara Y, Kanamori H, Kobayashi F, Sasaki H (2014). Genome-wide transcriptome analysis reveals that cadmium stress signaling controls the expression of genes in drought stress signal pathways in rice. Plos One.

[29] Zhang M, Liu X, Yuan L, Wu K, Duan J, Wang X (2012). Transcriptional profiling in cadmium-treated rice seedling roots using suppressive subtractive hybridization. Plant Physiol Bioch.

[30] Wang SQ, Dai HP, Skuza L, Chen YQ, Wei SH (2022). Difference in Cd2+ flux around the root tips of different soybean (Glycine max L.) cultivars and physiological response under mild cadmium stress. Chemosphere.

[31] Zhan J, Twardowska I, Wang S, Wei S, Chen Y, Ljupco M (2019). Prospective sustainable production of safe food for growing population based on the soybean (Glycine max L. Merr.) crops under Cd soil contamination stress. J Clean Prod.

[32] Zhang S, Song J, Wu L, Chen Z (2021). Worldwide cadmium accumulation in soybean grains and feasibility of food production on contaminated calcareous soils. Environ Pollut.

[33] Arao T, Ae N, Sugiyama M, Takahashi M (2003). Genotypic differences in cadmium uptake and distribution in soybeans. Plant Soil..

[34] Boggess SF, Willavize S, Koeppe DE (1978). Differential response of response of soybean varieties to soil cadmium. Agron. J..

[35] Codex Alimentrius Commission (2001). Report of the 33rd Session of the Codex Committee on Food Additives and Contaminants.

[36] Zhou Q, Guo JJ, He CT, Shen C, Huang YY, Chen JX (2016). Comparative transcriptome analysis between low- and high-cadmium-accumulating genotypes of Pakchoi (Brassica chinensis L.) in response to cadmium stress. Environ Sci Technol.

[37] Normanly J, Slovin JP, Cohen JD (1995). Rethinkin Auxin biosynthesis and metabolism. Plant Physiol.

[38] Quiroga M, Guerrero C, Botella MA, Barceló A, Amaya I, Medina MI (2000). A tomato Peroxidase involved in the synthesis of lignin and Suberin1. Plant Physiol.

[39] Martone PT, Estevez JM, Lu F, Ruel K, Denny MW, Somerville C (2009). Discovery of lignin in seaweed reveals convergent evolution of cell-wall architecture. Curr Biol.

[40] Chaoui A, Jarrar B, El FE (2004). Effects of cadmium and copper on peroxidase, NADH oxidase and IAA oxidase activities in cell wall, soluble and microsomal membrane fractions of pea roots. J Plant Physiol.

[41] Halušková LU, Valentovičová K, Huttová J, Mistrík I, Tamás L (2010). Effect of heavy metals on root growth and peroxidase activity in barley root tip. Acta Physiol Plant.

[42] Hernandez LE, Sobrino-Plata J, Belen Montero-Palmero M, Carrasco-Gil S, Laura Flores-Caceres M, Ortega-Villasante C (2015). Contribution of glutathione to the control of cellular redox homeostasis under toxic metal and metalloid stress. J Exp Bot.

[43] Clemens S, Kim EJ, Neumann D, Schroeder JI (1999). Tolerance to toxic metals by a gene family of phytochelatin synthases from plants and yeast. Embo J.

[44] Kim H, Yim B, Kim J, Kim H, Lee Y (2017). Molecular characterization of ABC transporters in marine ciliate, Euplotes crassus: Identification and response to cadmium and benzo[a]pyrene. Mar Pollut Bull.

[45] Kim DY, Bovet L, Maeshima M, Martinoia E, Lee Y (2007). The ABC transporter AtPDR8 is a cadmium extrusion pump conferring heavy metal resistance. Plant J.

[46] Moons A (2003). Ospdr9, which encodes a PDR-type ABC transporter, is induced by heavy metals, hypoxic stress and redox perturbations in rice roots. Febs Lett.

[47] Guerinot ML (2000). The ZIP family of metal transporters. Bba-Biomembranes.

[48] Plaza S, Tearall KL, Zhao F, Buchner P, McGrath SP, Hawkesford MJ (2007). Expression and functional analysis of metal transporter genes in two contrasting ecotypes of the hyperaccumulator Thlaspi calerulescens. J Exp Bot.

[49] Gill SS, Tuteja N (2014). Cadmium stress tolerance in crop plants: probing the role of sulfur. (Special Issue: Plant abiotic stress.). Plant Signal Behav.

[50] Swarup R, Bhosale R (2019). Developmenal roles of AUX1/LAX Auxin Influx carriers in plants. Front Plant Sci.

[51] Zhu FZ, Wang ZW, Dong F, Lei GJ, Shi YZ, Li GX (2013). Exogenous auxin alleviates cadmium toxicity in Arabidopsis thaliana by stimulating synthesis of hemicellulose 1 and increasing the cadmium fixation capacity of root cell walls. J Hazard Mater.

[52] Yu CL, Sun CD, Shen CJ, Wang SK, Liu F, Chen YL (2015). The auxin transporter, OsAUX1, is involved in primary root and root hair elongation and in Cd stress response in rice (Oryza sativa L.). Plant J.

[53] DalCorso G, Farinati S, Furini A (2010). Regulatory networks of cadmium stress in plants. Plant Signal Behav.

[54] Xue M, Zhou YH, Yang ZY, Lin BY, Yuan JG, Wu SS (2014). Comparisons in subcellular and biochemical behaviors of cadmium between low-Cd and high-Cd accumulation cultivars of pakchoi (Brassica chinensis L.). Front Env Sci Eng.

[55] Trapnell C, Roberts A, Goff L, Pertea G, Kim D, Kelley DR, Rinn JL, Pachter L. et al. Nat Protoc. 2012; 7: 562-578. 10.1038/nprot.2012.016 10.1038/nprot.2012.016 PMC3334321 22383036

[56] Anders S, Huber W (2010). Differential expression analysis for sequence count data. Genome Biol.

[57] The Genome Sequence Archive Family (2021). Toward explosive data growth and diverse data types. Genom Proteom Bioinf.

[58] Database Resources of the National Genomics Data Center (2022). China National Center for Bioinformation in 2022. Nucleic Acids Res.

